# Assessment of the Light Exposures of Shift-working Nurses in London and Dortmund in Relation to Recommendations for Sleep and Circadian Health

**DOI:** 10.1093/annweh/wxab092

**Published:** 2021-10-25

**Authors:** Luke L A Price, Marina Khazova, Ljiljana Udovičić

**Affiliations:** Radiation Dosimetry Department, Centre for Radiation, Chemical and Environmental Hazards, Public Health England, Harwell Campus, Chilton, Didcot, Oxfordshire, OX11 0RQ, UK; Radiation Dosimetry Department, Centre for Radiation, Chemical and Environmental Hazards, Public Health England, Harwell Campus, Chilton, Didcot, Oxfordshire, OX11 0RQ, UK; Federal Institute for Occupational Safety and Health (BAuA), Friedrich-Henkel-Weg 1-25, 44149 Dortmund, Germany

**Keywords:** circadian rhythms, healthcare, light exposure, night work, nurses, shift work

## Abstract

Shift work causes disruption to circadian physiological processes in the human body, and desynchronization from the natural day-and-night rhythm. Circadian disruption is thought to explain the associations between shift work and various long-term diseases; light is an unrivalled synchronizer (or Zeitgeber) of circadian processes and inappropriate light exposure plausibly plays a critical role in the development of health impairments. As published measurement data on the actual light environments encountered by shift workers are sparse, nurses working in two hospitals in London (UK) and Dortmund (Germany) wore light-logging dosimetry devices to measure personal light exposures continuously over a week in three different seasons. The study identifies and quantifies several of the characteristics of light exposure related to different working patterns in winter, spring, and summer, and quantifies interindividual variations. These data enable informed design of light exposure interventions or changes to shifts to reduce unwanted effects of disruptive light exposure profiles.

**What's Important About This Paper?**

This paper is important because it reports 24-hour light exposures of shift working nurses at comparable locations in the UK and Germany. Light exposures had high interindividual variation, and for many nurses were restricted and not representative of the solar profile. These data can guide design of interventions to improve the health of shift workers, including light interventions and changes in patterns of work shift.

## Introduction

The sleep–wake cycle, core body temperature, and the release of hormones such as melatonin and cortisol are physiological processes that follow circadian rhythms, meaning that they repeat with a period length of about 24 h. The timing of these circadian processes is orchestrated by a coordinating ‘central clock’ located in the hypothalamus (e.g. see Foster, 2020). In humans, and many other species, ambient light reaching the retina is the primary stimulus for synchronizing the clock to environmental changes over the day and night.

Shift work and night work may include working activity that starts or ends outside of daylight hours, and working hours that vary substantially on different days relative to the solar clock. Too great an exposure to light at night or not enough light during the day can contribute to circadian disruption, for example when working night shifts or when working predominantly indoors with little access to daylight, respectively (Ganesan *et al.*, 2019; Kang *et al.*, 2020). Long-term sleep and circadian rhythm disruption (Foster, 2020) is thought to explain the associations between shift work – night work in particular – and health impairments such as metabolic disorders, cardiovascular disease, and even an increased incidence of cancers (Straif *et al.*, 2007; Hansen, 2017; Wang *et al.*, 2018; Khosravipour *et al.*, 2019; Foster, 2020; Silva *et al.*, 2020).

When designing light interventions and working patterns to improve health, it is important to consider the light exposures resulting from current practice and anticipate what differences any interventions, including shift changes, are likely to bring about (Price *et al.*, 2019). With the latest knowledge of the effects of light on the circadian clock, and utilizing suitable wearable light-logging dosimeters, there is an opportunity to get a deeper insight than ever before.

In 2018, the International Commission for Illumination (CIE) recommended researchers and lighting professionals use a new standard metrology for light received by the eyes in relation to circadian rhythms and other ‘non-visual’ responses (CIE, 2018). The standard builds on metrology recommendations from an international consensus of independent experts in circadian and neurophysiological photometry (Lucas *et al.*, 2014). Melanopic Equivalent Daylight (D65) Illuminance (melanopic EDI; CIE, 2018) has been recommended as the practical measure for determining the likelihood and magnitude of non-visual responses relating to everyday exposures (Brown *et al.*, 2020). Melanopic EDI should be measured close to the eye ‘in the vertical plane’, in contrast to measurements of illuminance, which relates to illumination for the visual system and are typically carried out in the object plane, often on a horizontal working surface (e.g. Spitschan *et al.*, 2019). A minimum melanopic EDI during the daytime of 250 lx was also recommended (Brown *et al.*, 2020), along with a maximum of 10 lx in the 3 h before intended bedtime and further restrictions for the night. These recommendations were influenced by a recent meta-analysis of relevant laboratory studies (Brown, 2020), and the use of melanopic EDI is consistent with some earlier analyses and proposals (Price, 2014; Berman and Clear, 2019; CIE, 2019; Prayag *et al.*, 2019). Melanopic EDI of 4 lx to 300 lx is thought to represent the interquartile range of human responses to light in practically relevant conditions (Brown, 2020). A review of several recommendations relating to non-visual light exposures at workplaces is available (Stefani and Cajochen, 2021) but the reviewed recommendations are largely simplified by excluding from scope exposures from shift work, night work, commuting, and/or time outdoors.

There are relatively few results from similar field studies to date. Rabstein *et al.* (2019) reported 24 h blue light exposures for shift work in Bochum (51.5°N, Germany). There are melanopic EDI data for evening exposures at private homes in Melbourne (38°S, Australia), but not at work, not over entire days, and not for shift workers (Cain *et al.*, 2020). A related laboratory protocol was used to link these domestic measurements to biological factors and had shown that individual differences might frustrate one-size-fits-all recommendations (Phillips *et al.*, 2019), so understanding the causes of this variability would be useful. However, adequate sunlight exposure reduces interindividual variability of timing and amplitude in circadian rhythms (Wright Jr *et al*., 2013), adapting to different day lengths due to the seasons (Stothard *et al.*, 2017); both studies at 40°N, Colorado, USA. Therefore, minimum daytime thresholds combined with maximum evening thresholds may be an effective basic strategy for supporting circadian entrainment even when applied to groups of people simultaneously.

A recent office worker study at Alphen aan den Rijn (52.1°N, Netherlands), used the blue sensor from a LightLog dosimeter worn on the upper chest (van Duijnhoven *et al.*, 2021). These data may be used to derive melanopic EDI data (see Discussion). Similarly, unpublished data from domestic (at 51°N, UK) (Santhi *et al.*, 2012) and work-related (41-42°N, Chicago, US, Crowley *et al.*, 2015 and 55-56°N, Denmark, Daugaard *et al.*, 2019) light exposure studies should include sufficient information to derive usable melanopic EDI time-series (Price *et al.*, 2012).

Comparisons between studies continue to be hampered by the use of light-logging dosimeters often measuring inappropriate quantities, worn in different locations facing different directions, or by results being presented using non-standard terms. In order to understand the circadian light exposures at the eye relating to different working patterns and shift arrangements, this study looks at personal light exposures, measured continuously for a week (literally ‘24-7’), of nurses working in two hospitals in the UK and Germany, and across three seasons, with six different working patterns, using measurements of melanopic EDI, supplemented by illuminance measurements.

The aim was to present melanopic light exposure data relating to shift workers in northerly latitudes, that are useful to establish what types of interventions, if any, would be most appropriate to help with circadian and sleep health disruption.

## Methods

Altogether, 85 study participants (43 nurses from the Liver Intensive Treatment Unit at King’s College Hospital in London and 42 nurses in various roles at Klinikum Dortmund) took part in the study. The shift-working nurses in Dortmund worked in three shifts of approximately 8.5 h including 30-minute handovers and breaks (early, late, and night shifts starting at 06:00, 14:00, and 22:00 respectively). Those in London worked in two approximately 12.5-hour shifts including 30-minute handovers and breaks (long night and long day shifts starting at 07:30 and 19:30 respectively). Finally, the UK day-working nurses worked approximately 8 h (8 h days starting at 09:00), inclusive of breaks.

London and Dortmund lie on the same latitude (51.5°N), so the daylight durations in the two cities are identical. Sunrise, midday, and sunset occur approximately 30 min earlier in Dortmund than London. However, using local clocks the situation is reversed: if the sun rises in London at 06:00 GMT, the sunrise in Dortmund has already happened at approximately 06:30 CET. The approximate daylight hours during the measurement weeks were as follows: winter 8 h 40 min (London 07:50–16:30, Dortmund 08:20–17:00); spring 14 h 15 min (London 05:50–20:05, Dortmund 06:20–20:35); and summer 16 h 40 min (London 04:40–21:20, Dortmund 05:10–21:50).

Data were acquired for three separate weeks in January, April, and June 2015, at the same times in London and Dortmund, both during and outside working hours. Continuous 168-hour time series of the light exposure from natural and artificial sources were recorded per study participant and per season. 24-hour excerpts of the time series relating to different types of working patterns (days, shifts, or nights) were selected for analysis. Data from night shifts ran shift-to-shift on consecutive night shifts, whereas other working patterns ran sleep-to-sleep (before dawn). Exclusions were made if hours worked did not match the usual shift timings or if the light-logging dosimeter was not worn as required. Data based on days not working in the hospitals are not presented here, due to the widely varied patterns of activities, sleep, and compliance.

The nurses were recruited in person in collaboration with hospital (ward) administrators, from those expected to be regularly working during the subject periods. The study participants completed an initial questionnaire, an environmental questionnaire, and an activity diary. The initial questionnaire asked for general information, such as age and working pattern. The work and home environment questions asked about the type of lighting sources being used. In the activity diary, time spent indoors (in the hospital, at home, etc.), or outdoors (walking, sports activities, etc.) were recorded in half-hour segments, providing information on events that affected the light encountered. The data from the questionnaires, activity diary, and light exposure detectors were anonymized using a separate four-digit code for each nurse before being saved and analysed.

Light exposures were logged every 30 s using ‘AWS’ dosimeters (Actiwatch Spectrum, Philips Respironics) attached to clothing at chest height. In addition to actimetry capabilities (Murphy, 2009; Marino *et al.*, 2013), each AWS has three sensors that respond to light in the red, green, and blue regions of the visible spectrum, between 350 nm and 750 nm, and reports the irradiance measured by the sensors (R, G and B) as well as illuminance. Both illuminance and melanopic EDI data were combined into hourly averages in the local time zones, to obtain 24 hourly values for each of the six shift types in each season, for example the ‘average hourly’ value between 14:00 and 15:00 is assigned to 14:30. Each set of 24 average values is for all nurses’ shifts matching the (location × shift-type × season) description and may include multiple contributions from any individual nurse.

To assess the stimulus for non-visual responses to ambient light, spectrally-matched melanopic EDI data were calculated by combining the responses of the G and B sensor (Price *et al.*, 2012; Price *et al.*, 2017). The measurement performance of the AWS dosimeters was characterized in our optics laboratories, either in the UK or Germany (Price *et al.*, 2012; Udovičić *et al.*, 2016). The sensors were linear over several orders of magnitude, with negligible dark signals, and the spectral mismatch for the melanopic EDI data was approximately 16%. Illuminance measured with the AWS sensors has spectral mismatch *f*_1_’ ≈ 90%, and hence is only approximate (Price *et al.*, 2017).

## Results

In total, 595 working days were evaluated (395 in Dortmund and 199 in London, after exclusions for incomplete data). Fewer working days were evaluated in the UK, partly owing to the longer and less frequent shifts, and partly to the lower compliance in the UK during spring, when the workloads on the ward were frequently close to capacity.

Table 1 shows, for each shift-type and season, the London and Dortmund nurses’ average luminous exposure in terms of melanopic equivalent daylight exposure, in lx·h (melanopic EDE = average melanopic EDI × duration), between 08:00 and 20:00, subdivided into three daytime periods, and between 20:00 and 08:00, subdivided into two equal halves. 1000 lx·h and 3000 lx·h are equal to 4 h and 12 h at a melanopic EDI of 250 lx, the minimum daytime level recommended in Brown *et al.* (2020). The first daytime period in Table 1 is only 3 h, as this is often said to be the most important time for exposure (e.g. in Foster, 2020), and the last daytime period is 5 h to compensate.

**Table 1.** Average melanopic EDE (in lx·h) for nurses in different seasons, and different day periods. Daytime periods (08:00–11:00, 11:00–15:00, and 15:00–20:00) on average >1000 lx·h, or >3000 lx·h for the entire day, are shown in bold text (excluding nights and night shifts), and subtotals are shown on shaded rows.

London, UK	8-h day		Long day shift			Long night shift			
	Winter	Summer	Winter	Spring	Summer	Winter	Spring		Summer
08:00–11:00	215	**1724**	127	307	253	201	1131		1326
11:00–15:00	626	883	244	401	314	156	167		49
15:00–20:00	96	**1483**	120	230	301	44	649		1366
08:00–20:00	938	**4091**	491	938	868	401	1946		2741
20:00–02:00	25	54	50	49	145	60	57		78
02:00–08:00	8	751	31	264	409	26	73		78
20:00–08:00	32	805	81	313	554	86	130		156
Total, 24 h	970	4896	572	1251	1422	487	2076		2897
Dortmund, Germany	Early shift			Late shift			Night shift		
	Winter	Spring	Summer	Winter	Spring	Summer	Winter	Spring	Summer
08:00–11:00	150	337	470	124	**1212**	938	4	204	255
11:00–15:00	389	**1123**	**1395**	606	**3114**	**2133**	179	123	198
15:00–20:00	186	**2492**	**4190**	150	782	785	129	2374	1987
08:00–20:00	725	**3952**	**6056**	880	**5108**	**3857**	312	2701	2440
20:00–02:00	10	20	85	64	79	159	133	129	240
02:00–08:00	54	102	253	2	67	29	86	224	371
20:00–08:00	64	122	338	66	146	188	219	353	611
Total, 24 h	789	4074	6394	946	5254	4044	531	3054	3051

**Figure 1.**
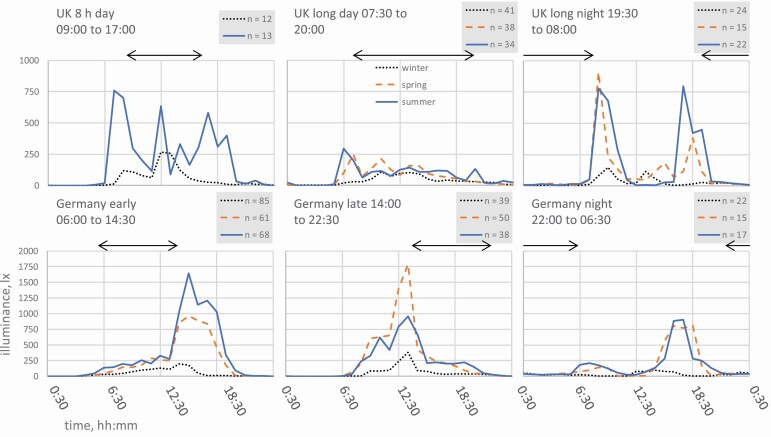
Average hourly illuminance over 24 h in London, UK and Dortmund, Germany in winter (dotted line), spring (orange dashed line), and summer (blue solid line), for six distinct shift types. Arrows indicate shift times at the hospitals. Only night shifts following night shifts 24 h earlier are included, to isolate conditions encountered between successive night shifts. n represents the number of separate 24 h time series combined for each shift type and season.

**Figure 2.**
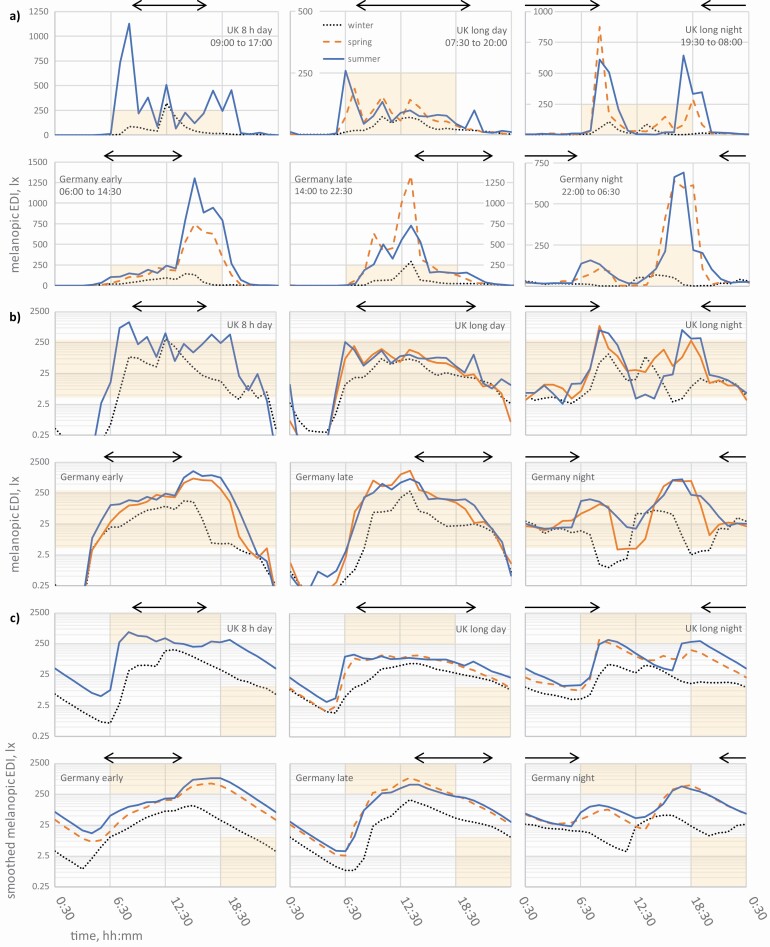
a) Average hourly melanopic EDI in lx (layout and key as Fig. 1). The area of each grid box represents a melanopic EDE = 1500 lx·h (melanopic EDI = 250 lx for 6 h) or half the proposed minimum daily melanopic EDE = 3000 lx·h (shaded area). b) The same data as a) but using a fixed vertical log scale. The interquartile range of human responses to light, melanopic EDI of 4 lx to 300 lx, in practically relevant conditions (Brown, 2020) is shaded. c) The same data and scale as b) after smoothing with a 90 min half-life (Price, 2014). A time series passing through a shaded region indicates either a smoothed melanopic EDI > 250 lx in the daytime or < 10 lx in the evening.

The illuminance data were presented, along with blue light irradiance, that is from the B sensor alone, in a comprehensive project report (Udovičić *et al.*, 2020), which also included information on the types of lighting reported by the nurses. The average hourly illuminance time series include large intra- and inter-individual differences, but work and seasonal dependencies are clearly recognizable in both London and Dortmund when the individual participants’ data are combined. The combined average hourly illuminance profiles for the different shift types are reproduced in the six panels of Fig. 1, for comparison with the melanopic EDI profiles shown in Fig. 2a. The working times are also indicated in Figs. 1 and 2.

Melanopic EDI (e.g. Fig. 2a) tended to be lower than illuminance (Fig. 1) during working hours (indoor periods), but it was similar when daylight was the dominant condition. The changes at low illuminance levels when switching to melanopic EDI are obscured on a linear vertical scale; the fixed log scale for melanopic EDI (e.g. Fig. 2b) better supports most comparisons. Finally, smoothed melanopic EDI in Fig. 2c is based on a simplified moving average model with a 90 min half-life to account for the reductions in efficacy with exposure duration and the post-illumination persistency of nocturnal responses (Chang *et al.*, 2012; Price, 2014). The model is designed to estimate the effective stimulus from variable light exposures. Under a constant exposure, the model gradually converges to the constant melanopic EDI, from a previous state defined by the recent prior light history.

Figs. 1, 2a and b are presented as arithmetic averages. Substantial variations in the hourly light exposures arise due to different activities between individuals and different weather and lighting conditions between days. The distribution of hourly light exposures between individual nurses is typically skewed, with the standard deviation greater than the average; it is not clear what statistical model should apply, and there is no established or agreed method.

To quantify the interindividual variations of the melanopic EDI series shown in Fig. 2, the half-width of the 95% confidence interval (CI) of hourly light exposures for a log-normal distribution was calculated (e.g. Crowley *et al.*, 2015). For a given combination of shift and season, the CI half-width typically varies between 0.6 and 3.3 orders of magnitude (0.6 to 1.3 during working hours) depending on time of day and location, as well as shift and season. For the smoothed melanopic EDI these CI half-widths reduce to between 0.5 and 1.5 orders of magnitude (0.5 to 0.8 during working hours).

Seasonal differences can also be seen; in winter, the total daytime melanopic EDI for all working patterns was lower than in spring and summer, apart from night workers during the period 11:00–15:00 (see Table 1). The change in the duration of daylight at different times of the year was reflected in the changed melanopic EDI in the late afternoon and early evening on early shift working days in Dortmund (Fig. 2b).

The data presented are based on combining isolated shifts, but the sequence in which shifts and recovery days are performed and combined with free time and study time was highly variable. For example, Table 2 shows sequences worked, and confirmed with actimetry, during the first visit for several of the UK nurses.

**Table 2.** Shift sequences for several nurses in London, separated by semi-colons. An individual nurse may contribute no more than 7 complete days, in one or two comma-separated sequences. Commas indicate a period without compliant light exposure and actimetry data reconciled to the diary. Bold text indicates a contribution to the light exposure data analysed.

Shift sequences FF**DD**FF, **D**F; F**D**F; F**D**_**8**_**D**N, FD; D**D**FF; D_10_NO; NFFFN**NN**; FF, N**N**F; FF**DD**, FFF; N**N**; D**D**F, FF; N**N**N; OOOO, N**N**; D_9_**D**NFN; NFON, NFF; D_8_D_5_D_17_R, F**D**_**8**_D_5_D_8_; D_8_**D**_**8**_D_8_, F**D**_**8**_**D**_**8**_D_8_; DFD, D**D**; D_8_N_4.5_N, N**NNN**_**2**_FD; F**D**F**D**FFD

Notes on data included for further analysis: A bold **N** indicates the second shift from a 24 h shift-to-shift period. **D**_**8**_ in bold indicates compliant 8 h days that start at approximately 09:00. First and last sequence days in plain text are non-compliant or incomplete (<24 h available), or were not shifts days.

D/N = long day/night, D_x_/N_x_ = day/night work for *x* hours, F = free or recovery time, O = other/study. D_17_ indicates a 5-hour day followed by a long night in the same 24-h period and N_2_ a night shift which ended prematurely.

### Day-active work

This section deals with the 8 h day and long day shifts in London, and the early and late shifts in Dortmund. The locations will only be repeated when needed. The data in Table 1 are given in bold when over 1000 lx·h for one of the three daytime periods, or above 3000 lx·h for the three periods combined. The conditions often fell short of these levels, and the charts in Fig. 2 can be used to understand the light profiles in further detail.

The ambient light conditions encountered were heavily influenced by the working hours (Fig. 2b), and the ambient light indoors from artificial lighting sources and daylight at work was considerably dimmer than from daylight outdoors. For all shifts in spring and summer, melanopic EDI only reached the upper quartile of non-visual responses to light (i.e. reached above the shaded band) during the hours immediately before or after work, due to the increased daylight exposure usually associated with journeys to or from the hospital. In winter, only the late shift reached this level, doing so at 13:00–14:00.

The melanopic EDI threshold of 250 lx can be applied to the smoothed time series model described above, as shown in Fig. 2c. Under this approach, long day workers did not reach the 250 lx target in any season, nor was it met in winter for any day-active shift type. The long day shifts (07:30–20:00) rule out meaningful daylight exposure on any day of the year, if, as here, no breaks are taken outdoors. Maintaining this working pattern over a few days could contribute to circadian desynchronization and disturbance of the sleep–wake rhythm.

In spring and summer, the late shift workers and 8 h day (summer only) reached the revised 250 lx target, on the average, before their working hours, and early shift workers only after their working hours. This highlights that exposure to daylight during journeys to and from work has a key influence on work-related exposures to light.

Average hourly ambient light conditions at the eye only dropped into the lowest quartile of non-visual responses to light (i.e. <4 lx) either after 22:00, or at approximately 19:00 for the early shift in winter (Fig. 2b). It is unclear what caused the average summer conditions throughout the night to be barely below this level for the long day workers. Although the 8 h days and the early shift were below or at 10 lx for three hours in the evening, this was observed only for these work patterns and only in winter. In contrast, pre-bedtime conditions below 10 lx do not appear to be naturally observed by workers on early and late shifts in spring and summer (or the long day shift in summer), and certainly not for the recommended three hours in Brown *et al.* (2020).

A positive finding for the late shift in Germany was the high average melanopic EDI achieved before working hours (14:00–22:30), as morning light exposure is important for synchronizing to the day-and-night rhythm. Indeed, this shift’s smoothed light profile closely resembles the regular 8 h day workers, especially when compared to either the long day or early shifts, suggesting it could also support a day-active circadian synchrony, possibly with a slight phase delay.

### Night shifts

This section concerns the 8.5-hour night shift in Dortmund (22:00–06:30) and the long night shift in London (19:30–08:00). All the exposure data are from 24 h periods ending with a night shift after a previous night shift. For both shift types, there were two maxima of average hourly exposure, in the early morning immediately after work and in the afternoon, also outside of working hours (see Fig. 2b). The first peak was roughly equal to the second for the long night shift, but less pronounced for the 8.5-hour night shift as travel home happened almost 2 h earlier in terms of solar time. Thus, winter lowered morning daylight exposure preferentially in Germany, changing the overall pattern of exposure for both locations, by also advancing and lowering the second peak.

High daylight exposure on the way home after the end of the night shift is undesirable, as it can impair the quality of sleep and recovery (Czeisler and Dijk, 1995), so this difference may be important. Fig. 2c shows that, apart from the winter profile after the 8.5-hour night shift in Dortmund, the smoothed melanopic EDI after work remains much higher than the 10 lx maximum recommended in the 3 h before sleep (by Brown *et al.*, 2020). In addition, night work naturally also leads to long nocturnal light exposures during work, albeit at low-moderate levels on average for these workers (melanopic EDI of 10 lx to 25 lx).

## Discussion

### Limitations of this study

This is an observational dosimetry study and the presented data do not include biological outcome variables (other than outcomes reflected in the light exposure itself), so the results and discussion do not directly analyse the associations between the light exposures and health, but consider the exposures against existing and possible recommendations for health based on past analyses only (including Brown, 2020; Brown *et al*., 2020).

The data presented only relate to three specific weeks and two locations with only the existing shift patterns and behaviours. They illustrate an often under-appreciated point – how varied light exposure influences are in different contexts. Rather than being a comprehensive study of light exposure and shift work, this study aims to provide data to guide designs of interventions intended to modify light exposures in future studies.

The data are presented in terms of group averages by shift type or working pattern, but the data are not randomized, and variances are not presented as these may not be sufficiently reproducible. Comparisons between different shifts and seasons should be treated with appropriate caution, and for greater confidence would need to be confirmed using a randomized study including weeks from each season repeated over many years.

### On recommendations for day-active workers

The recommended minimum melanopic EDI of 250 lx applies to the daytime (Brown *et al.*, 2020). However, over and above this minimum, it is not yet clear what the appropriate daily duration nor time-course of exposure should be, and the recommendations may become more detailed with further research. One way to assess variable daytime exposure profiles is considered further below, and this is followed by a similar discussion for the Brown *et al.* (2020) recommended evening and the night-time thresholds.

Living with modern lighting tends to delay circadian phase, so morning exposures are usually recommended for circadian entrainment in day-active people. Secondly, the saturation of responses for increasing durations shown in Chang *et al.* (2012) relates to corneal illuminance of approximately 9000 lx using 4100 K fluorescent lamps (melanopic EDI ≈ 5200 lx, based on our estimate of the ratio of melanopic EDI/illuminance or M/P ratio ≈ 0.58, see Schlangen and Price, 2021). This suggests that a greater positive daytime impact may be derived from separate periods of higher intensity light than a longer consolidated period of constant light with a given melanopic EDE. Thirdly, robustness of circadian entrainment may be achieved by exposure to bright light towards the middle of the day; one study showed increases in circadian rhythm amplitude following bright light administered in the daytime in otherwise healthy individuals with initially reduced circadian amplitudes (see Fig. 5 in Jewett *et al.*, 1994). Other studies showed that the adverse effects of evening and nocturnal light on sleep quality, melatonin levels, and delays in circadian phase in healthy individuals could be lessened by exposures to additional light delivered at some point during the day (Munch *et al*., 2016; te Kulve *et al.*, 2019).

There are also potential added benefits of a few bright light exposures, with melanopic EDI >> 250 lx, distributed periodically throughout the subjective day-time, rather than strictly adhering to a continuous 250 lx minimum, provided the same average exposure is achieved and the exposure is not back-end loaded (i.e. biased towards the end of the day). This could then be achieved in different ways, and not require changes to increase lighting directly surrounding patients, for instance, and the smoothed melanopic EDI could be used to assess the adequacy of resulting exposures (Price, 2014). Provided it could be accessed, daylight would be available whenever required, except in winter, and as few as three exposures of 1000 lx·h distributed appropriately during the daytime working hours might be sufficient.

The findings of Munch *et al*. (2016) and te Kulve *et al.* (2019) also show that absolute measures of light cannot predict the nocturnal responses, and suggest an alternative approach to the evening and possibly the night-time recommendations in Brown *et al.* (2020). It follows that fairly simple relative measures of light might perform better than absolute thresholds. For instance, rather than imposing a 10 lx maximum, one could specify that the smoothed melanopic EDI (see Fig. 2c) should follow a minimum hourly rate of decrease in the 3 h before bedtime, although such an approach would need further validation.

A day-time exposure schedule may not be universally appropriate, for example for night shift nurses who work during the hours of darkness (Andersen *et al.*, 2012) or workers and patients needing to sleep during daylight hours.

### On recommendations for night shifts

For shift work, no simple advice about exposure to light applies to all scenarios (Price *et al.*, 2019). The phases of both activity that is supported by light, and sleep that should ideally be in darkness (Lucas *et al.*, 2014; Brown *et al.*, 2020), conflict with the natural pattern of daylight and the social environment. The separation between activity and sleep phases may also be compressed or the sleep phase fragmented. Our own data illustrate diverse scheduling arrangements for providing 24 h hospital nursing, for example Table 2. The phase-mismatches just described were all evident in both UK 12:12 and Germany 8:8:8 shift pattern data, and the light profiles in Fig. 2 demonstrate how the phases of light are also shifted. For the shifts containing night work, including the early shifts in Germany, the new light phase does not match the work activity phase. Additionally, the light exposure may be fragmented into two periods either side of a shortened sleep window. Through the physiological effects of light, the exposures from commuting in daylight hours after the end of the shift can place further restrictions on this opportunity for sleep.

Whereas for daytime work in both workforces, light interventions at work (possibly daylight breaks of 15 min) during the three daytime periods are indicated for winter, and during the 11:00–15:00 period throughout the year, daytime light interventions may not be relevant, for UK night shift workers. Here an intervention that may be advisable is the use of goggles to block blue light during the commute home.

Rescheduling is itself a class of intervention. This study does not speak to the effects of shift schedules on efficiency and error rates or accidents at work and whilst driving to and from work. If these are likely to be significant enough on their own to justify revising the approach to shift patterns, any change would still entail considering a range of options. Aside from changes for errors and accidents, the data suggest one option in the UK is for a 2-hour advance of the hand-over period between the long day and night shifts aimed at reducing the impact on subsequent sleep of natural light exposure during the commute after long night shifts, and increasing the daytime exposure following long day shifts. Evidence for the intended reductions in light exposure this would have is clear from the Germany data, although sunrises in Dortmund are 30 min later relative to local clocks. There are numerous further practical occupational and social differences to weigh up, but the revised long day shifts would still include the hours of 8 h day workers, who typically started work at around 09:00. An 8:8:8 pattern based on this 05:30 handover time would be another option.

### Rotations and scheduling

When rescheduling night shifts, the use of rotations, changes to rotations, and the impact of any changes on the physiology of workers doing other shifts and during their own time should be considered. Day-active people entrain to day length due to the season (Stothard *et al.*, 2017), and can re-entrain to exclusively natural light exposures in just a few days. For other shift patterns, it is not clear how many days are needed to re-entrain circadian rhythm and sleep timing to a (shift type × exposure) combination, or to recover natural entrainment following a series of consecutive shifts. In this regard, it was notable that Figs. 2b and c suggest the differences in exposures from different shifts could be of comparable or greater biological significance than the differences between winter, spring, and summer. The findings of Stothard *et al.* (2017) not only confirm the importance of considering different day lengths and latitudes separately, as here, but helps to emphasize the relative importance of considering shift sequences in future work.

Moving the underlying circadian phase of individuals to match the shift-pattern phases would, in theory, support health. However, this entails separating workers from the social advantages of maintaining a day-active phase (as closely as may be possible) and requires a level of shielding from natural daylight that in most cases is impractical. Workers would effectively live in an unnatural bubble, largely isolated for long periods from the wider world, friends, and family, and not without further problems. For instance, the sufficiency of light for activity may be restricted (e.g. for inpatients’ sleep in the case of their overnight nursing), and the transition to the required shift-pattern phase is likely to take several days. The potential long-term health impact of regularly undertaking phase shifting, of 8 h say, itself is not well known.

### Recent studies

The reported blue-sensor irradiance, ‘*B*_day_’, collected by office workers in Alphen aan den Rijn (52.1°N, Netherlands) (van Duijnhoven *et al.*, 2021) can be converted to melanopic EDI, assuming that blue-sensor irradiance is approximately equal to melanopic irradiance. On this basis, the mean reported melanopic EDE for these office workers’ on working 8 h on average including breaks in May and June was approximately 5800 lx·h, with highest irradiances before and after work and during lunchtimes. Compared to 4091 lx·h for the summer average melanopic EDE UK 8-h day workers, the difference largely results from lower average outdoor exposures after the UK working days.

The 17.9 lx median melanopic EDI for evening domestic indoor exposure in Melbourne, Australia (Cain *et al.*, 2020), is consistent with the light measured by the nurses in the hospitals overnight, with the lower level of 10 lx on average in the Intensive Treatment ward in the UK being similar to the lower quartiles in Melbourne homes.

The night shift nurses’ blue light exposures at work inside another hospital in Bochum, Germany (also at 51.5°N) were also similar in magnitude (Rabstein *et al.*, 2019), but the maximum geometric average daytime exposures away from work appear to be appreciably lower than the arithmetic average melanopic EDI data in this study (>250 lx in spring and summer). It can be seen from Fig. 2b that these levels are similar between both locations here, but that they vary due to season. Geometric averaging explains some of the difference, but averaging exposure profiles across seasons removed information that is necessary for determining the potential effects on sleep.

For one shift type, night shift nurses’ 24 h blue light exposures, when averaged across different day lengths, were observed to be bimodal (Rabstein *et al.*, 2019). This might result for combining two separate modalities, say from two seasons, or from a bimodality phenomenon that persists down to the level of the individuals’ exposure in one 24-hour period. The melanopic EDI data here confirms that this bimodality can be observed from averages within seasons, because bimodality is present in the underlying individual data, and applies to differing extents for two more types of night shift and locations, varies according to day length, changing qualitatively in winter, and hence we can confirm that the primary cause for the observed bimodality is exposure to daylight during commuting to and from work.

## Conclusions

Photometric measurements of light in controlled laboratory environments were adequate to unravel the basic framework of the problems with sleep, health, and shift work some time ago (Czeisler and Dijk, 1995). Our melanopic light exposure field data can strengthen that evidence base and are intended to be used in the design of interventions as well as to supplement emerging epidemiological data concerning associations between light exposures due to shift work and a widening array of adverse health outcomes.

However, the full width of the 95% confidence interval between log-transformed hourly light exposures for nurses at work in the same hospital on the same shift is around two orders of magnitude. The need and potential to optimize the light exposure at work is therefore likely to vary substantially between individual nurses, and the situation away from work further increases interindividual variations.

Light exposure advice taken in isolation cannot be a panacea for shift workers; at best, it is hoped that good advice in the future will help mitigate, rather than eliminate, the health consequences associated with regularly undertaking shift work. Even after all reasonable steps to minimize the health impacts associated with shift work have been taken, it is important that employees are given enough information to allow for informed decisions about the remaining risks, even if they will not usually be entirely free from influence owing to their personal circumstances.

Most importantly, our data and analysis confirm that 24-hour dosimetry data are needed to build up an accurate picture of the light exposure associated with a given shift work arrangement. These data prevent advice and interventions relating to light exposure and scheduling from being inadvertently at odds with key influences on the circadian responses to light and the implications for healthy sleep.

Access to sufficient daylight combined with a stable day-night pattern of light exposure is widely recognized as a basic need for ensuring human health and wellbeing. There are now recommendations on what 24-hour light profiles best support the synchronization of the circadian system, and should be considered most beneficial to health, although these are still vague in terms of the interactions between the timing, intensity, and the durations of realistic variable light exposures. The light exposure profiles in this study are variable, but clearly show that, for many nurses, exposures to daylight on working days are restricted and not representative of the solar profile.

Travel to and from work in daylight hours plays a significant disruptive role for the sleep and day-active circadian rhythms of night shift workers. The extent of these effects depend on the shift schedules, solar phase in local time, and day lengths as well as the duration and mode of travel. Reducing exposures of post-work commuting for night shift workers may have significantly higher impact than workplace light interventions and should be considered in future study designs. Long daytime shifts in London also substantially reduced the hospital workers’ exposures, to well below recommended levels. In Dortmund, exposures for the 8.5-hour work patterns that included daytime work were also substantially skewed towards exposure outside of work hours in spring and summer. The hospital lighting provided nurses with exposures in the night and evening after sunset that were comparable with those measured in the evenings in private homes. The data collected can support the designs of light interventions and further research to minimize the adverse impact of working various shift patterns on sleep and circadian rhythms.

## Data Availability

The data underlying this article are available on request from the corresponding author.
