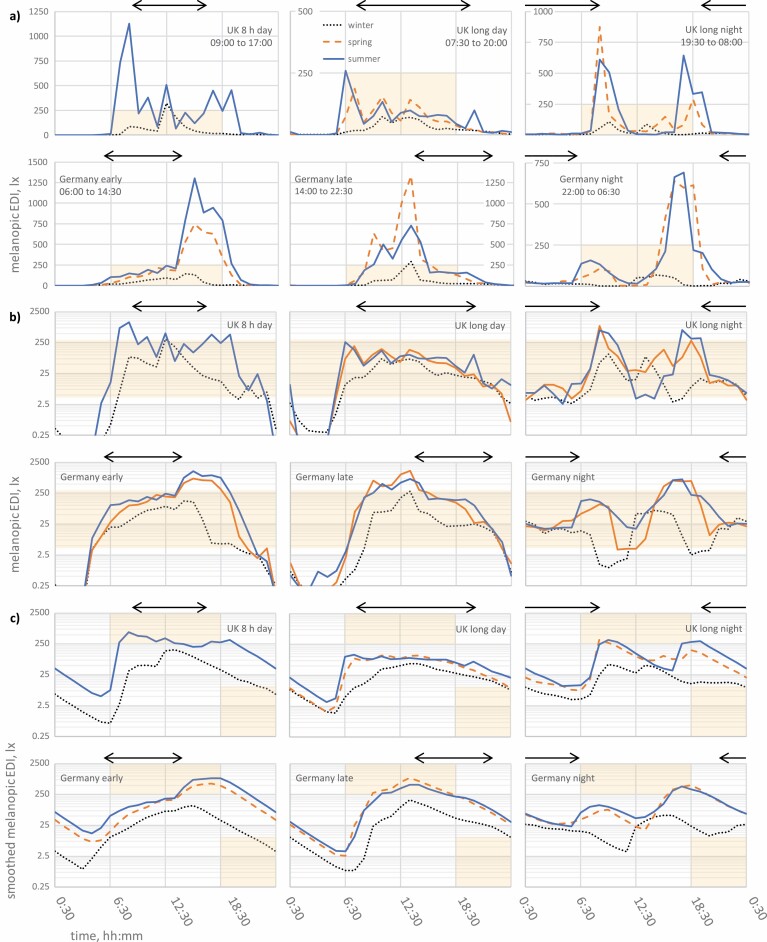# Erratum to: Assessment of the Light Exposures of Shift-working Nurses in London and Dortmund in Relation to Recommendations for Sleep and Circadian Health

**DOI:** 10.1093/annweh/wxab109

**Published:** 2021-11-26

**Authors:** Luke L A Price, Marina Khazova, Ljiljana Udovičić

**Affiliations:** 1 Centre for Radiation, Chemical and Environmental Hazards, Public Health England, Harwell Campus, Chilton, Didcot, Oxfordshire, UK; 2 Federal Institute for Occupational Safety and Health (BAuA), Friedrich-Henkel-Weg 1-25, 44149 Dortmund, Germany

Due to production error, critical shading errors were inadvertently introduced to data presented in Figure 2. The below stated error was only present in the originally published PDF version of this article and has since been corrected online. The Publisher would like to apologize for the error and any inconvenience caused to the reader. 

Previous version:


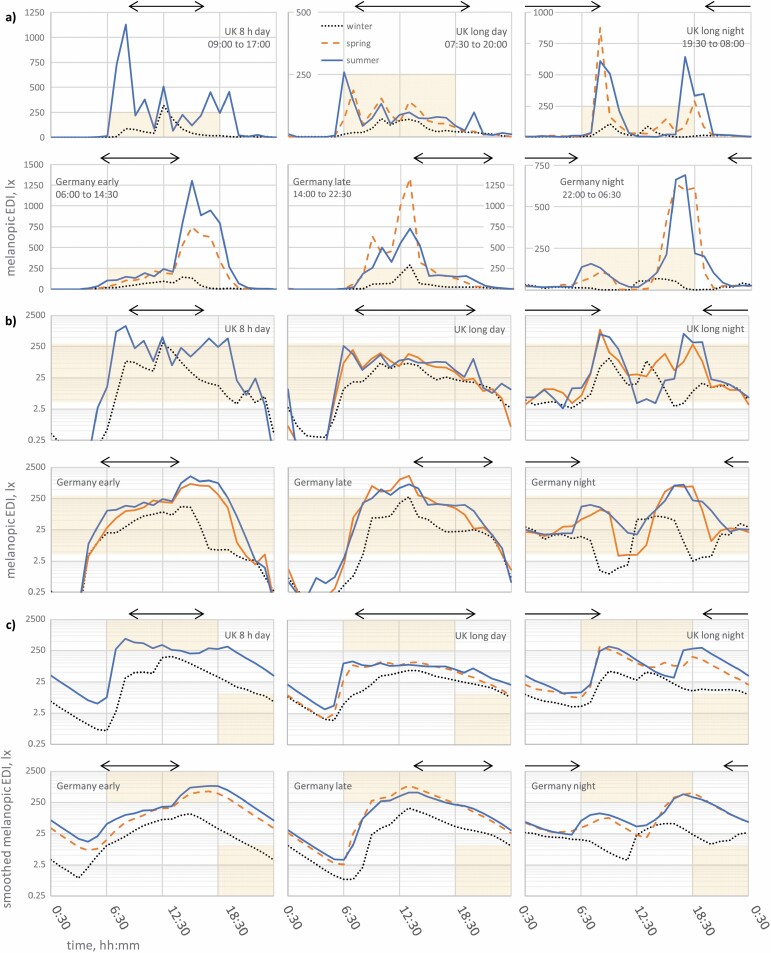


Corrected version: